# Happy Children: A Modern Emotional Commitment

**DOI:** 10.3389/fpsyg.2019.02025

**Published:** 2019-09-06

**Authors:** Peter N. Stearns

**Affiliations:** Department of History, George Mason University, Fairfax, VA, United States

**Keywords:** happy children, childhood, emotions history, American culture, childrearing, happiness

## Abstract

American parents greatly value children’s happiness, citing it well above other possible priorities. This commitment to happiness, shared with parents in other Western societies but not elsewhere, is an important feature of popular emotional culture. But the commitment is also the product of modern history, emerging clearly only in the 19th century. This article explains the contrast between more traditional and modern views, and explains the origins but also the evolution of the idea of a happy childhood. Early outcomes, for example, included the novel practice of hosting parties for children’s birthdays, another mid-19th-century innovation that has expanded over time. Explaining the intensification of the happiness commitment also reveals some of the downsides of this aspect of popular emotional culture, for example in measurably complicating reactions to childish unhappiness. The basic goal of the essay is to use this important facet of modern emotional history to evaluate a commitment that many modern parents assume is simply natural.

One of the most pervasive beliefs about emotion, at least in American culture, is the idea that children should be happy and that childhood should be a happy, perhaps unusually happy, stage of life. There is little question that many parents are strongly guided by this standard, even though a variety of experts argue that they often go about it in the wrong way. And it is highly likely that many adults simply assume that childhood happiness is a natural connection, that while its implementation may be varied and debated and while a few reprobates may not accept the goal at all, the basic notion is simply a normal part of human life.

International polling confirms the pervasiveness of the happy childhood assumption, in American and several other cultures – though it also opens the door for a somewhat more nuanced assessment. A recent survey found that 73% of Americans rated happiness as the most important goal in raising children and assessing the results of education – far ahead of any other option. And they were joined, or even modestly surpassed, by a number of other modern Western societies: Canada at 78%, with France heading the pack at 86%. Other goals paled in comparison, even though it was possible to select more than one option: only 20% of Americans rated success as a major goal (along with 17% in Australia and the United Kingdom).

However – and here is the first opening for more than a brief summary of the happiness/childhood emotional linkage – several other major societies presented quite a different profile in the same poll. Most strikingly only about 49% of respondents in India selected happiness, overshadowed by the 51% who put success and achievement first. Mexicans also rated success most highly. The Chinese, interestingly, did not seize on success but they did not highlight happiness either, putting good health at the top of the list. The poll suggested, plausibly enough, that a predominant commitment to children’s happiness was an artifact of advanced economic development (bolstered, quite possibly, by a particular dose of Westernism as well) (Malhotra, 2015).

Certainly the American assumption that happiness and childhood go together can be additionally confirmed. A childrearing expert, Robin Berman, puts it this way: “When I give parenting lectures around the country, I always ask the audience ‘What do you want most for your children/’… The near-universal response I get is ‘I just want my kids to be happy.”’ Berman herself deeply challenges the validity of this commitment, but for now the main point, again, is to emphasize the depth of the American assumption (shared, clearly, with other Western societies). It is understandable that many Americans simply take the priority for granted, open perhaps to a discussion of what strategies best achieve the goal but not inclined to subject the goal itself to much scrutiny. The idea that children should be happy, indeed that childhood stands out as a particularly happy time of life, is deeply ingrained (Berman, 2016).

But without placing too much emphasis on international polling, the gap between Western and Asian (or Mexican) responses already suggests that the childhood/happiness equation is not automatic or in any sense natural, but the product of more particular circumstance. And this in turn opens the way to a more searching analysis, aimed initially at determining where the idea that children should be happy came from in the first place and then tracing the way the association has evolved in the United States, with some clear downsides or problems attached.

Assessing the childhood/happiness linkage provides in fact a fruitful opportunity to demonstrate the role of emotions history in shedding light on significant popular assumptions and commitments. The emotions history field, which has grown rapidly within the history discipline over the past 30 years, contends that key aspects of the emotional beliefs and experiences of any society are shaped not by invariable psychobiology but by particular social and cultural circumstances. This means that we can learn more about the past by including emotional variables in the human equation and that – as in this case – we can understand current patterns better if we examine how they have emerged from contrasting assumptions in the past (Matt and Stearns, 2013; Boddice, 2018).

In the case of happy children, the emotions history approach raises two initial questions, before we get into most recent evolution of the association: what did people think about happiness and childhood at an earlier point and when (and of course why) did the happiness emphasis begin to develop.

The most glaring historical challenge to the childhood happiness equation is not easy to handle, but it adds up to the statement: before about the middle of the 19th century most Americans (and, probably, most people in most agricultural societies) did not equate children and happiness and indeed were unlikely to see childhood as a particularly happy phase of life (Greven, 1988; Mintz, 2006). This does not mean that they necessarily expected children to be unhappy, or that they were gratuitously nasty to children, or that they did not enjoy moments of shared joy. But any kind of systematic happiness, or even a common use of the term, was simply not part of popular expectations (Gillis, 1981).^1^

And the reasons for this stance are not hard to identify, in a combination of general features of premodern childhood and some particular cultural assumptions that took deep root in colonial America. In the first place, high child mortality rates – with 30–50% of all children born perishing before age 5 – surrounded children themselves with frequent death and constrained adult reactions as well. A dead child might be deeply mourned, but the expectation of transiency obviously affected perceptions of childhood more generally: adulthood could easily be seen as a preferable state. Further, for most people childhood after infancy was primarily associated with work, under the sometimes rough direction of adults. Childishness, in this context, was not highly valued, as opposed to the early acquisition of more mature qualities. In all probability, obedience was the quality most sought in children themselves. Small wonder that, before the 19th century, few autobiographers spent much time describing their childhoods in any detail or referring to their early years with any pleasure (Stearns, 2016).

This is not to say that before the 19th century children had no pleasure, or that adults never enjoyed their more informal interactions with offspring: considerable historical debate cautions against too gloomy a view. Work requirements were not always too intense, particularly for younger children, and there were informal opportunities for playfulness (Huizinga, 2016).^2^ Traditional leisure outlets, and particularly the village festival, gave young people some space for pranks and hijinks. But none of this seriously qualifies the claim that more systematic ideas associating childhood with happiness were lacking.

In the colonial American context, this general situation was exacerbated, particularly in New England, by the strong Protestant commitment to the notion of original sin. How many adults viewed actual children through this severe lens is hard to determine, though it was certainly linked to harsh disciplinary practices in schoolrooms and churches. But even if youngsters were not actively seen as sinners requiring redress, Protestant beliefs certainly argued against conceptions of happy childhoods. Indeed a number of studies suggest that, even for adults, an emphasis on a degree of melancholy was urged even for adults, well into the 18th century (Greven, 1988; Demos, 1999; Mintz, 2006).

Granting the perils of trying to establish the absence of a quality in the past, the claim seems reasonably secure: the association of childhood and systematic happiness, as opposed to periodic moments of release, is essentially a modern development.

Several factors, taking shape in the later 18th and early 19th centuries in the United States and other parts of the Western world, began to reshape the conception of childhood, despite the lingering hand of the past.

Interest in happiness in general began to accelerate in Western culture during the second half of the 19th century (Kotchimedova, 2005; MacMahon, 2006; Jones, 2017). The Enlightenment encouraged a new commitment to optimism about life on this earth, and hopes for happiness increased accordingly. Apologies for good humor, common during the previous century with its preference for melancholy in the face of human sinfulness, began to disappear (Stearns, 1988). Even more, a positive expectation that decent people should present a cheerful demeanor began to gain ground. One historian has suggested that, along with the general push from Enlightenment thinking, improvements in dentistry and a decrease in rotten teeth heightened a willingness to smile openly – and to expect others to do the same (Jones, 2017). Emphasis on happiness may also have been furthered by some measurable improvements in life’s comforts, from home heating to cleaner clothing, at least for the property-owning middle classes. And of course, in revolutionary America, pursuit of happiness was listed as a basic right.

This significant cultural shift did not initially apply to children, at least with any specificity. Older beliefs persisted. Checking the rise of attention through the relative frequency word use (happiness, cheerfulness) bears this out suggestively (Figures 1, 2). Google Ngrams suggest the chronological lag: while references to cheerfulness and happiness in general peaked in relative frequency during the 18th century in American English, commentary on happy children was virtually non-existent until the 19th century, and became at all common only in the middle decades of the century.^3^

**FIGURE 1:**
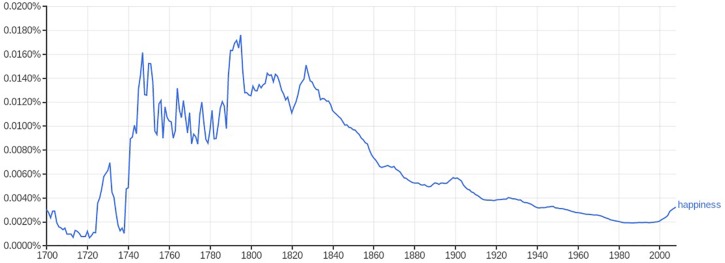
Frequency of the word “happiness” in American English, 1700–2008, Google Ngram viewer, accessed March 19, 2019.

**FIGURE 2:**
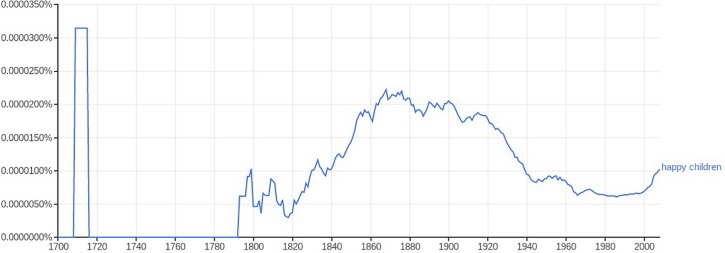
Frequency of the phrase “happy children” in American English, 1700–2008, Google Ngram viewer, accessed March 19, 2019.

Obviously, sometime was required to overcome earlier assumptions and extend new cultural expectations downward in age. For several decades after 1800, some halting steps suggested the difficulty of fully overcoming earlier standards: thus the idea of cheerful obedience gained ground in family manuals. Insistence on obedience was maintained, but for the first time the potentially demanding hope that it could be accompanied by a cheerful demeanor was added to the list (Stearns, 2014).

In addition to the time required to apply an initially adult innovation to the thinking about children, several other shifts in the first half of the 19th century further explain the timing of the change. Most obviously, amid intense American debate during the 1820s and 1830s, traditional notions of original sin were increasingly replaced, in mainstream Protestantism, by insistence on childish innocence. By the late 1820s the most widely purchased family manuals routinely highlighted children’s sweetness and purity, which only bad adult behavior would corrupt. An obvious barrier to the notion that children might be happy was being lifted, though amid ongoing sectarian dispute (Sedgwick, 1850).^4^

Here it is possible to see how the new cultural commitment to happiness combined with several other social factors to generate a new approach to children. Most obviously the birth rate began to drop, which may have facilitated more emotional attention to the individual child. Work obligations increasingly yielded to schooling as a child’s obligation, in the middle classes; seeing children in less functional terms might have contributed to a new interest in happiness, bolstered as well by a desire to cushion the burdens of education. Most tantalizingly, the middle decades of the 19th century saw a general middle-class interest in portraying the family as an emotional refuge from the complexities of economic and social life amid early industrialization – what one historian has called the family as “haven in a heartless world.” Here was a clear context for new attention to cheerful children as part of this equation, linking the shift to social pressures as well as the larger cultural framework (Lasch, 1977; Mintz, 2006).

Certainly, as the role of the middle-class family began to shift away from function as a production unit and toward service as a source of emotional refuge and support, the ideal of a loving and happy assemblage, children very much included both as beneficiary and source, became increasingly common. While smile-drenched photographs would await the 20th century, in part thanks to improvements in technology, positive representations of the family unit, often grouped around a piano, became increasingly common (Mintz, 2006).

Again, the middle decades of the 19th century were something of a transition. Association of happiness and childhood continued to gain ground, but explicit discussions of parental obligations concerning happiness, or of happiness as an explicit goal, were not yet fully developed. Had polls been conducted in the 1850s, they might have suggested the same kind of priorities for achievement or health, over happiness, that remain common in places like India or China today.

But one innovation, that would ultimately become emblematic of the conversion of expectations about childhood, quietly started becoming standard fare in middle-class life: the (presumably) happy birthday – directed toward children – girls and boys alike – above all. Here is another case – more specific than children’s happiness in general – where understanding innovation cuts through any assumptions of timelessness (Pleck, 2000; Baselice et al., 2019).

For children’s birthdays are a modern invention. Royals publicly touted their birthdays in many societies, going back to the Egyptian pharaohs, as a means of promoting public attention and support. European aristocrats may have begun celebrations in the 18th century, but the emphasis was on adults as well as social privilege. The idea of singling out children depended on a much higher valuation of their individual importance than any traditional society had generated – which is why the emergence of the new practice is so revealing.

The first recorded child birthday in what was becoming the United States occurred in Boston in 1772, for the 12-year old daughter of a wealthy family. Presumably this was a way to show off the family’s wealth as well as honoring a child. As the birthday practice began to spread, very slowly, several goals were often mentioned besides the family’s material achievement: a means of encouraging young people to display gratitude and sometimes as well an opportunity for the birthday child to give little gifts to servants as a token of appreciation (Pleck, 2000; Cross, 2004).

By the middle of the 19th century birthday celebrations were clearly becoming more common. Several manuals were written to guide the practice, one of them going through several editions. Emphasis rested on a modest party, with pastries and special fruits (commercial baking was improving at this point, thanks in part to German immigration: an obvious source of cakes). Parents would usually offer a single gift, sometimes a toy but sometimes religious or educational material. By the 1870s, when the hugely successful *Ladies Home Journal* was founded, women’s magazines began to feature stories about successful birthdays at least once a year, until (by 1900) the practice had become so common that guidance was no longer needed (except perhaps for encouraging parties for adults as well). By this point many African American schools were also celebrating birthdays, and there were signs of working-class and immigrant interest as well (Prentiss, 1857; Barnard, 1861; Leslie, 1869; Industrial School for Colored Girls, 1916).

The new practice faced some opposition (as it still does today in societies were birthdays are just beginning to surface). Some religious writers worried that children would be made too prideful, that a celebration that should actually honor God, or at least one’s parents, was being distorted. While worries about consumer excess were not yet common (this would await the 20th century), some commentators criticized children who came to insist on annual festivities; the demanding child was hardly a traditional ideal (Davenport, 1864; Hill, 1906).

But, obviously, birthdays advanced quite rapidly, clearly indicating an eagerness to highlight the individual child, and this even before the massive reduction in child mortality that would further support the practice. And the question, briefly, is why. Of course families imitated each other; undoubtedly children learned from their friends and put some quiet pressure on their parents; consumer success and opportunities to display gratitude continued to enter in. But by the 1850s all the published recommendations on birthdays, and all the comments from approving parents, stressed the role of these festivities in making children happy.

The parents and prescriptive writers who commented on birthdays and cheered them on made the basic goal very clear: birthdays were becoming important because they made children happy, and happiness in turn was quietly turning into a priority. Thus a comment in 1886 insisted that birthdays should be pleasurable, full of “rejoicing jubilees”: “a ripe, full year is a glorious thing to have had,” and for their part children, “poor little things,” “need all the fun they can get.” Schools began to pick up the celebratory theme: a Helena, Montana, high school noted “the charming custom” growing among students and teachers to acknowledge the occasion through surprise parties and small gifts. Late 19th-century etiquette writers, recommending birthday festivities, urged the occasions be “joyous, for children are easy to please” and “nothing is quite as beautiful and gratifying as a group of laughing, happy children.” Childrearing manuals, though late to the topic, echoed similar sentiments. Right after 1900 Alice Birney commended regular attention to birthdays by “makers of happy homes” because of the “pleasure and enthusiasm” that the festivities generated (A New Idea, 1855; Aldrich, 1891^5^; Gardner, 1904; Primary Education Journal, 1907; Buffalo High School Yearbook, 1925; Helena Independent Newspaper, 1982).^6^

Beyond the rise of the birthday and its signal connection to aspirations for children’s happiness (and the concomitant expansion of Christmas celebrations), wider commentary on the importance of happy childhood proliferated in the early 20th century. Whereas 19th-century childrearing manuals had remained somewhat hesitant, prioritizing other goals and insisting on connecting happiness to moral behavior, popular entries after 1900 prioritized the goal with no strings attached. “Don’t forget to be indulgent; do your best to make a pleasure possible, and enter heartily into it.” To be sure, parental “readiness” to “bring happiness into your children’s lives” should be rewarded by good behavior. But happiness began to be its own goal, predicated on a belief that children’s dispositions prepared adult qualities, and was important to train people up to be cheerful (Leach, 1993).

From about 1915 on, the happiness theme became truly ubiquitous. “Happiness is as essential as food if a child is to develop into normal manhood or womanhood.” Parents had a “duty” to make their offspring happy: “The purpose of bringing-up in all its phases should be to make the child as happy as possible” (italicized in the original for emphasis) (Birney, 1905). “Make a child happy now and you will make him happy 20 years from now… And happiness is a great thing…It contributes to the making of a normal childhood, which is in turn the foundation of normal manhood or womanhood.” Chapters of parenting books began to be devoted explicitly to the need to promote childish happiness, even, in many accounts, as the expense of discipline. Even the rather severe behaviorist, John Watson, intoned, “Failure to bring up a happy child…falls on the parents’ shoulders” (Stearns, 2012). And, symbolizing the intensification, it was in the 1920s that the song “Happy Birthday” emerged, gaining widespread popularity during the following decade. Enjoyment and nurturing of happy children had become a central feature of ideal family life but also a solemn obligation as part of preparing for successful adulthoods. Finally, the theme began to spill beyond family life, to other institutions that dealt with children. “Cheerfulness” was one of the twelve characteristics enshrined in Boy Scout Law, for example, while the Campfire Girls insisted on happiness directly. And – though this issue remains with us today – schools and teachers began to be drawn into concerns about children’s happiness as well (Groves and Groves, 1924; Spalding, 1930; De Kok, 1935; Baruch, 1949; Gruenberg, 1968).

Intensification of the childhood/happiness has obviously continued into recent decades, among other things adding measurably to parental obligations. By the 1960s parents were reporting an increasing sense of obligation to play regularly with their children, as part of their commitment to sponsoring happiness. In the schools, the Social and Emotional Learning movement (another 1960s product) has gained ground, urging teachers to emphasize positivity and guard against less happy emotions. Serving the happy child continues to gain momentum (Stearns, 2019).

But the main point – happy childhood as a product of recent history – deserves primary emphasis. The commitment to happy childhoods obviously builds on the precedents that had developed during the later 19th century. It connected quite explicitly to increasing hopes for happiness in life in general and to beliefs that cheerful people were more likely to win success in life. And the escalation surely benefited from the new demographic framework: with low birth rates and, now, rapidly declining child mortality, it was easier to connect the early years of life with more positive goals. Happy childhoods became part of what has been aptly described as the rise of the “priceless” child (Zelizer, 1994).

Though the idea of children’s happiness emerged over time, and responded to a number of wider cultural and social changes, it must be remembered that it was a really new aspiration. The fact that most modern American, or French, or Canadian parents regard it as a normal goal, indeed a self-evident priority, should not disguise its innovative nature or, in historical terms, its relative recency. Our current assumptions have a past, responding to a changing environment.

But there is more to this historical perspective as well, including some complexities that are at least as relevant to contemporary childhood and parenting as the happiness commitment itself. The evolution of the idea of the happy child, particularly from the early 20th century onward, also highlights some of its downsides and risks. Three points stand out, all of which add to the expansion of parental obligations inherent in the modern happiness theme itself: the extent of parental responsibility: the association with consumerism; and, above all, the problem of sadness.

The first wrinkle in the surge of interest in children’s happiness, as it took shape from the early 20th century onward, was a basic question that was, however, rarely hauled out for explicit evaluation: were children naturally happy, or did parents (and other adults) have an obligation to create happiness in a more difficult terrain? Commentary on birthdays in the 19th century occasionally, as we have seen, suggested that the celebration should help compensate for a less-than-joyous stage in life. And this might touch base with more traditional ideas about the drawbacks to being a child. On the other hand, enthusiasm about childish innocence, though more modern, might emphasize children’s spontaneous gaiety and their positive contribution to a cheerful family.

Actual childrearing materials frequently suggested a mixed opinion – sometimes within a single passage. Thus from a 1920s manual: “childhood is meant to be a joyous time. In the opinion of most adults it is actually the most joyous time of life” (the dramatically modern view). But then, twenty lines down, “Nevertheless it is the province and duty of parents to make the childhood of their progeny a joyous time.” Other materials suggested that the obligations here could be quite demanding.”: “Avoid unpleasant incidents like the plague. They shake the fabric of happiness to its foundations.” Make sure that kids never go to bed sad: “Darling we are quite happy now, aren’t we? Look up and smile at mother… You know she loves you so much and wants you to be always the very happiest little boy in all the world”(O’Shea, 1920; Galloway, 2013).

Inconsistency about children’s nature, where happiness was concerned, may be built into the modern process to some extent. Many parents will have days when they can simply capitalize on a child’s good mood, and others when a tremendous amount of effort is involved. The uncertainty obviously staked out a potentially challenging obligation for adults, adding to the growing emotional list of what a good parent was responsible for: if children were not naturally happy, or when their mood turned sour, the vigilant parent needed to compensate. But uncertainties also spilled over into the other main complexities of the growing commitment to happiness.

This in turn relates to the second complexity. It was probably inevitable that interests in happy childhood became deeply connected with family consumerism. The marriage began to take clear shape early in the 20th century and it steadily intensified thereafter. The first explicit parental purchases for children date back to the late 18th century, when the focus was on the new genre of children’s books. Interest expanded in the 19th century, as in the practice of birthday gifts, but the range remained rather modest. But with the 20th century, and particularly with the rise of the toy industry, the interest in using purchases to promote children’s happiness became increasingly entrenched.

Many aspects of this intertwining are familiar enough. Shortly after 1900 many parents began to buy toys even for infants (including the soon-famous Teddy Bear). There was brief discussion of whether this kind of attachment to things was desirable in the very young, but hesitation was brief and short-lived. “Things” made children happy and prepared a life of consumer attachments, and they helped fulfill the otherwise daunting parental task of linking childhood and joy. Whole companies devoted their attention to the happiness connection: Disney, founded in the 1920s, made happiness its core theme, and later would proclaim that child-centered parks like the California Disneyland were the “happiest places in the whole world.” Not to be outdone, soon after World War II McDonalds would sell its child-focused and highly caloric burger combination as a “happy meal,” complete with cheap toys (Cross, 2004).

Another post-World War II innovation pushed the linkage further. Many parents began to prepare for Christmases or birthdays by encouraging their children to draw up wish lists, which usually turned out to be quite long and detailed exercises in maximization (Moir, 2017). The result? Another dilemma. As one children’s consumer expert put it: “how much do you want your child to be happy – meeting what you think are their desires?” (Rosen, 2015). Against this, the sheer limits of a family budget (though sometimes transcended through the credit card) and a recurrent concern that many kids were becoming too greedy and materialistic, that they were internalizing the happiness/consumerism equation too thoroughly. Worst of all was a growing belief that children learned, if unwittingly, to play on their parents’ commitment to happiness, developing a sense of entitlement that overwhelmed any sense of gratitude (Stearns, 2012).

The consumer/entertainment/happiness combine played on one final later 20th-century development: a redefinition of boredom. Boredom was a modern concept in itself: the word came into common usage only in the mid-19th century, associated obviously with the growing interest in active happiness. Initially, however, boredom applied to childhood mainly as a character lesson: children should be taught not to be boring. After 1950, however, the meaning was flipped: boredom now became a state to be blamed on others, a reason for personal discontent. And children became adept not only at identifying their boredom, but at strongly implying that their parents, or teachers, or others had an obligation to do something about it. “I’m bored” became yet another way of telling the adult world that it was falling short, for the child should be entertained (Stearns, 2003; Toohey, 2011).

In real life, of course, most children learned to handle a bit of moderation. Wish lists were rarely fully fleshed out, and children could even survive the lack of the year’s most popular toy or game. But the dedication of part of childhood to early forms of consumerism, and the pressure on parents to fulfill part of their happiness obligations through toys and entertainments, played no small role in actual family life and, sometimes, a nagging sense of falling slightly short.

And this linked to the third complexity of happy childhoods: the inevitable tensions that resulted when confronted with the unhappy child. Not surprisingly, the relative frequency of discussing unhappy children went up rather dramatically in the 19th century (as Google Ngrams suggest), as a counterpart to the new expectations more generally. While rates dropped a bit thereafter, the topic remained vivid, encouraged by growing interest in, and claims by, child psychologists and other experts. Two outcomes seem pretty obvious. First, of course, the unhappy child (or the period of unhappiness), whether directly experienced or not, was a cautionary tale for parents themselves: something must have gone wrong, some adult must have failed in her duties, for this to have emerged. The facile association of unhappy childhoods and parental dereliction (and often, resultant unhappy adulthoods) became a conversational and literary staple by the mid-20th century, particularly amid the popularization of Freudian psychology (Ludy, 2007). And second, when the unhappy child was encountered there was a risk of exculpatory diagnosis: the child must be unhappy because of some psychological disorder, the unhappiness a sign of some kind of illness, beyond the responsibility of good parents. It became harder to accept or even understand the sad child (Berman, 2016).

Historical value judgments are never easy, particularly since by definition we are trapped in our own contemporary standards. It is hard not to believe that, for all the complexities involved, the emergence of the idea of happy children was an advance over earlier frameworks – which is one reason that the idea of children’s happiness has spread geographically as part of globalization (though without yet creating uniform agreement). But, inevitably, since we are enmeshed in the happiness culture it is hard to evaluate it against past patterns.

Certainly, there are the downsides, which the historical approach, cutting through any assumption that the idea of happy childhood is a natural human concept, helps highlight as well. It becomes too easy to overdo the happiness card, whether the result is undue accumulation of childish junk or the difficulty of appreciating periods of childish sadness. It is easy to complicate the actual achievement of normal happiness by expecting too much, by reacting to quickly to emotional lows. As it emerged from the 19th century onward, the assumption that children should be cheerful as part of the child’s contribution to the happy family can be genuinely burdensome, just as the assumption places obligations on parents as well. The realization that much of this is a recent historical product, which might be open to some reconsideration or modification, can be constructive. Not a few experts are joining in urging greater nuance and flexibility about the childhood/happiness association.

There is one final point. We began this essay by noting the premium that Western parents, when polled, place on children’s happiness. But of course happiness is not the only thing we want, and it is even possible that our cultural standards prompt us to claim a higher priority than we really mean. Contemporary Americans certainly do not want unhappy children, but the classic helicopter parent, this creature of the past quarter century, may actually be more focused on achievement than we explicitly recognize – however, parentally orchestrated that achievement may be. Recent analysis that suggests how successful many middle-class parents have become in positioning their children for college and beyond, in a newly demanding economic environment, may complicate the happiness equation: these parents want to think their offspring are happy, but they are orchestrating other goals (Druckerman, 2019). The extent to which middle-class American parents are unusually focused on the importance of hard work, compared to European counterparts, certainly raises some questions about actual priorities, despite lip service to the hope for childish joy (Doepke and Zilibotti, 2019). The happiness standard will surely prompt the demanding parent to bursts of indulgence, often with a strong consumer component, and probably some real guilt about not succeeding as consistently on the happiness front as we would like.

The relatively modern conversion to the notion that children should be happy added important criteria to the ways many American parents evaluated their own performance and clearly helped motivate changes in actual interactions with children, including the growing commitment to consumerism. It affected people’s evaluations of their own childhoods, and could affect children directly as well, as in the injunctions to be cheerful. But, as several recent studies of happiness suggest, the results in terms of actual happiness and well being are harder to assess: expectations could be raised beyond reasonable hope of fulfillment, and signs of occasional sadness might become harder to handle (Ahmeds, 2010). Add into this the pressures for achievement and success, so vivid in the current generation of middle-class teenagers, and the evaluation of actual outcomes, as opposed to professed goals, becomes undeniably complicated.

## Author Contributions

The author confirms being the sole contributor of this work and has approved it for publication.

## Conflict of Interest Statement

The author declares that the research was conducted in the absence of any commercial or financial relationships that could be construed as a potential conflict of interest.

## Footnotes

1 In contrast, youth, though never systematically empowered, might be valued. Greek and other cultures celebrated the beauty and athletic prowess of youth. Artisanal arrangements in many societies – Europe, Japan, the Middle East cherished a few relatively carefree years between apprenticeship and full adulthood. But childhood was a different matter.

2 Huizinga’s *Homo Ludens* mounts a particularly detailed case for children’s play in traditional societies.

3 Google Ngram Viewer is a search application that allows one to measure the relative frequency of particular terms or words in the Google Books database. While in some ways problematic and obviously not a complete representation, the tool is a helpful way to assess cultural trends and changes.

4 These early Victorian manuals interestingly combine the emphasis on childish innocence with continued insistence on obedience with rarely if ever a bow to happiness.

5 Aldrich also writes of taking “personal note” of each student on the birthday.

6 As noted above, the *American Journal of Education* had been recommending birthdays since the 1860s.
